# Advancement in the Cuffless and Noninvasive Measurement of Blood Pressure: A Review of the Literature and Open Challenges

**DOI:** 10.3390/bioengineering10010027

**Published:** 2022-12-24

**Authors:** Mohammad Mahbubur Rahman Khan Mamun, Ahmed Sherif

**Affiliations:** 1 Department of Electrical and Computer Engineering, Tennessee Technological University, Cookeville, TN 38505, USA; 2 School of Computing Sciences and Computer Engineering, The University of Southern Mississippi, Hattiesburg, MS 39406, USA

**Keywords:** cuffless blood pressure, hypertension, photoplethysmography, electrocardiogram, machine learning, deep learning

## Abstract

Hypertension is a chronic condition that is one of the prominent reasons behind cardiovascular disease, brain stroke, and organ failure. Left unnoticed and untreated, the deterioration in a health condition could even result in mortality. If it can be detected early, with proper treatment, undesirable outcomes can be avoided. Until now, the gold standard is the invasive way of measuring blood pressure (BP) using a catheter. Additionally, the cuff-based and noninvasive methods are too cumbersome or inconvenient for frequent measurement of BP. With the advancement of sensor technology, signal processing techniques, and machine learning algorithms, researchers are trying to find the perfect relationships between biomedical signals and changes in BP. This paper is a literature review of the studies conducted on the cuffless noninvasive measurement of BP using biomedical signals. Relevant articles were selected using specific criteria, then traditional techniques for BP measurement were discussed along with a motivation for cuffless measurement use of biomedical signals and machine learning algorithms. The review focused on the progression of different noninvasive cuffless techniques rather than comparing performance among different studies. The literature survey concluded that the use of deep learning proved to be the most accurate among all the cuffless measurement techniques. On the other side, this accuracy has several disadvantages, such as lack of interpretability, computationally extensive, standard validation protocol, and lack of collaboration with health professionals. Additionally, the continuing work by researchers is progressing with a potential solution for these challenges. Finally, future research directions have been provided to encounter the challenges.

## 1. Introduction

More than a billion people suffer from hypertension in the whole world [1]. Hypertension increases the risk for life, endangering cardiovascular issues and severe diseases such as stroke, renal and kidney failure, etc. If unnoticed and not managed, hypertension can lead to serious health complications and may eventually deteriorate to the patient’s death. If monitored, hypertension and hypotension can be managed using lifestyle modification, change in food habits and medication suggested by a physician [2,3]. The problem is, unlike many major diseases in humans, abnormally high or low blood pressure may go unnoticed due to a lack of monitoring and lack of significant symptoms. The best way to detect this issue is to regularly monitor using a reliable method and keep track of the changes [4,5]. In every clinical guideline, hypertension and hypotension management constitute significant sections; for example, systolic blood pressure over 130–140 mmHg is categorized as stage 1 hypertension [6]. Diastolic blood pressure (DBP) below 60 mmHg and systolic blood pressure below 90 mmHg is called hypotension, which means below normal blood pressure (BP). Hypotension inflicts oxygen deprivation on different parts of the human body; tissue death happens because of that. Hypotension symptoms include but are not limited to dizziness, general pain, sluggishness, etc., which may end in severe consequences such as cardiac arrest; on the other hand, hypertension symptoms span from fatigue and shortness of breath to heart attack, stroke, and organ failure [7,8]. Prehypertension starts with SBP between 121–139 mmHg and DBP between 81–89 mmHg [9]. Although this is not staged one hypertension, prehypertension is within a range with the potential risk of going over to stage 1. SBP between 140–159 mmHg is hypertension 1, 160–179 mmHg is hypertension II, and over 180 mmHg is called hypertensive crisis [10,11,12,13,14]. During hypertension, the stress over the heart increases; although medical treatment may not be mandatory at this stage, frequent monitoring is necessary for a sudden spike during the daytime. During hypertension II, without monitoring and timely treatment, the higher pressure may result in organ failure and irreversible damage to different body parts. The hypertensive crisis may result in numbness of body parts, vision problems, breathing difficulty, etc. Eventually, a heart attack or stroke may occur if left untreated.

The research revolving around monitoring BP mainly follows two approaches; one is to try to make the existing cuff-based measurement system more user-friendly or adjustable to day-to-day life; another is to use biomedical signals which are easily accessible to find a technique that will measure BP within reasonable accuracy. Since the first approach’s accuracy level is already reasonable, making it smaller, more efficient, and robust are the main objectives of newer devices. In the second approach, reliability or accuracy is the biggest concern and versatility in terms of age and other factors. So, that approach emphasizes those since acquiring the needed biomedical signals already helps to keep those designs smaller while removing the necessity of external stimulations such as pressure on the artery. The advancement of sensor technology, signal processing techniques, PCB design, and artificial intelligence heavily influences the second approach. Stephen Hales did his horse’s first blood pressure measurement using an invasive method [15,16]. Later, the process was improved by physicians for human use while keeping the technique invasive [17]. The first catheter was implanted in 1949 into the artery of humans; this technique allows us to measure the BP recording without further discomfort [18]. Later this catheter technique was further improved by another scientist so that a wide variety of blood pressure could be observed [19,20,21]. In terms of accuracy or access to the wide variation of change in BP, the invasive technique is superior to all other techniques, but unless the patient requires urgent attention or in case of a routine checkup, this method is not feasible due to risk for infection, insertion discomfort, specialist to perform the procedure, blood loss and structural damage, etc. [22,23,24]. Because of those reasons, the research for noninvasive techniques progressed further in the initial period based on the cuff-based method, widely used at home and health centers [25,26,27,28,29]. Although the cuff-based method became less cumbersome due to the invention of a user-friendly device, the discomfort or the necessity of the cuff is not removed. That introduced the possibility of using other means to measure blood pressure while keeping the accuracy and reliability the same as before with conventional methods. Currently, a massive amount of medical data is available for data analysis; at the same time, the advancement of sensor technology allows us to acquire vital signs with precision and ease. Additionally, physiological data such as electrocardiography (ECG) and photoplethysmography (PPG) can be used for biomedical signal analysis [30]. All this can be brought together to invent new techniques or methods to measure blood pressure in a noninvasive and cuff way with the application of artificial intelligence [31,32,33,34,35]. Although theoretically, it is possible on paper since the data acquisition, design, signal processing, machine learning, etc., all are readily available to implement. However, till now, none of the techniques have become a reality with a reliable, accurate, and robust device which can replace the conventional technique.

### 1.1. Current Literature Survey

Several review papers have been available related to cuffless blood pressure measurement. Some of them focus on features from the PPG signal [36,37]; on the other hand, some encapsulate human physical activities to correlate them with BP variation [38,39]. A review on cuffless blood pressure monitoring has been done in this article [40], focusing on the necessity of measuring BP more frequently and continuously. The review also discussed the new sensors and algorithms incorporating machine learning. Another survey was done to incorporate the progress of narrow to deep learning methods for BP measurement while comparing the performance of PPG and ECG [41]. They have also compared different feature extraction techniques using PPG signal and included heart rate estimation along with BP. Shenda Hong et al. [42] discussed the opportunities and challenges of deep learning while using ECG signals for diagnosis purposes; during their discussion, they also discussed the possibility of using ECG signals to detect blood pressure measurement in a short subsection. One of the better literature surveys was done by Ramakrishna Mukkamala et al. [43], which encompasses cuffless blood pressure as a whole, including but not limited to historical methods, current cuff-based methods, and the research involving PPG, ECG signals. They have divided the study into calibrated and non-calibrated methods where features from PPG and ECG were put together into the calibrated method and use of oscillometric, volume control, and ultrasound into the uncalibrated method. Although other than PPG and ECG signals, there are other biomedical and physiological signals available in which researchers tried to get any relationship with the change of BP, one such literature review [44], which looked at all kinds of physiological signals, was done by Manisha Sharma et al. Ramakrishna Mukkamala et al. have done a review [45] emphasizing the cuffless wearable device and smart devices to measure blood pressure in the current market; they have also discussed a large number of publications or research happening where the conclusions are unreliable due to inadequate methodologies. In this review article [46], the future use of artificial intelligence in managing hypertension has been discussed; they have tried to examine the explainability of artificial intelligence (AI) to help health professionals understand the black-box nature of models. Kazuomi Kairo introduced the discussion [47] about using a miniature wearable monitoring device for remote measurement of BP; his primary focus was out-of-office BP measurement expecting dramatically change in the quality of hypertension management. Niklas Pilz et al. published a review [48] article discussing pulse wave velocity-related techniques for BP measurement and techniques involving deep learning to shortlist the advantages and limitations of different methods. Apart from these reviews above, some other literature surveys have been done [10,49,50,51] focused on ambulatory blood pressure monitoring, diagnosing hypertension, noninvasive techniques of BP measurement, contactless BP monitoring, etc.

### 1.2. Survey Goal

All the literature above focused on one or more aspects of BP measurement, from traditional invasive methods to current research involving noninvasive-cuffless techniques. The existing surveys still have some limitations, such as: using a limited number of studies or techniques and failing to cover both ECG and PPG signals to extract features for BP measurement. However, with the ever-evolving nature of technology with signal processing techniques and artificial intelligence, there are scopes to contribute with a holistic literature review on BP measurement, adding significant scientific value. We have presented the literature survey in the following manner: first, a framework for the search and selectin process has been provided; second, conventional BP measurement techniques are summarized; the third, motivation behind the cuffless BP measurement techniques was discussed; fourth, we have also brought forth the technologies which are helping to get us from traditional method to cuffless BP measurement, fifth, the features which have been used for techniques involving artificial intelligence were discussed, sixth, all the machine learning and deep learning techniques which have been used for BP measurement in recent years are summarized, seventh, the available datasets were summarized, and finally the challenges and future way forwards were proposed so that AI can have more significant impact finding techniques for reliable and robust BP measurement. The two main questions we have tried to solve in this literature survey are: (1) How are the standard features from biomedical signals used in current studies to find noninvasive and cuffless BP measurements? (2) How have artificial intelligence techniques (machine learning and deep learning) been proposed in recent research works for the same purpose, and which research directions need attention? To answer these research questions and understand the premise, we have covered traditional BP measurement techniques, cuff-based techniques, and the eventual use of AI for cuffless BP measurement.

## 2. Framework for the Search and Selection Process

**Figure 1:**
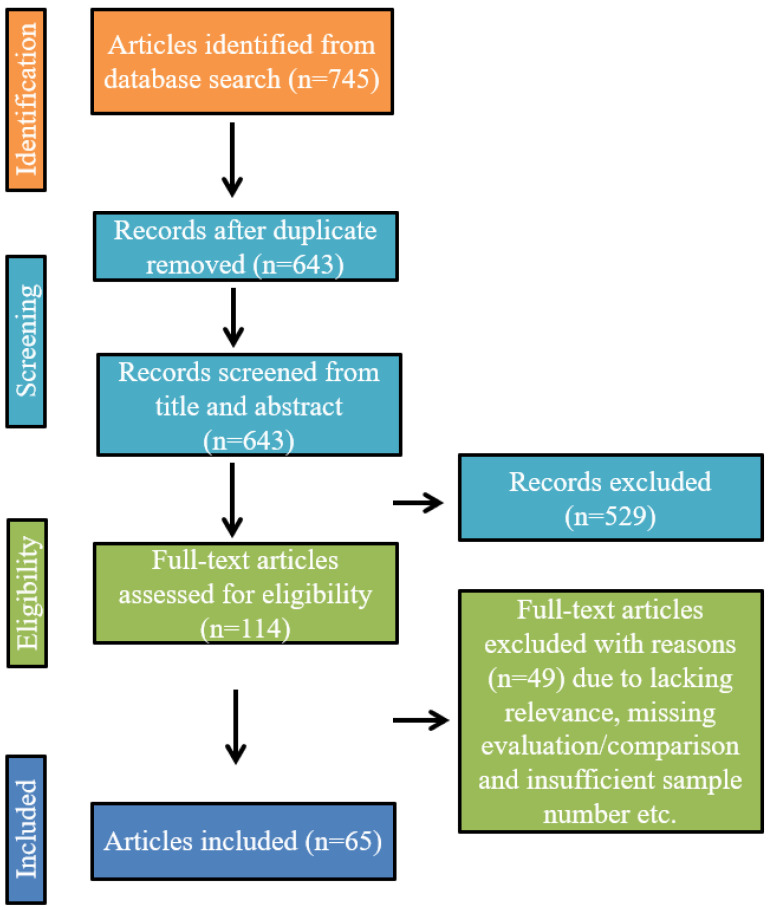
Flow diagram of the article selection process for literature review.

To incorporate a thorough review, the search procedure was implemented using machine learning and deep learning with features from biomedical signals to generate a model for measuring cuffless noninvasive BP. For this purpose, google scholar and PubMed were used with a timeline from 2010 to 2022. The following general search term was used from both database: (“ECG” or “electrocardiogram,” “ECG” or “EKG” or “PPG” or “photoplethysmography” or “biomedical signal,” “vital signs” or “physiological signals” or) and (“cuffless blood pressure” or “noninvasive blood pressure” or “hypertension” or “blood pressure” or “arterial pressure” or “hypotension” or “BP”) or (“machine learning” or “deep learning” or “artificial intelligence” or “neural network” or “convolutional neural network” or “ANN” or “CNN” or “long short term memory” or “LSTM” or “RNN” or “recurrent neural network”) or (“pulse transit time” or “pulse wave velocity”). It is essential to point out that using those keywords, many articles were found that were not related to this literature survey, and those were sorted out at the initial stage. Figure 1 depicts the selection process, divided into four stages (identification, screening, eligibility, and included). Overall, 745 articles were retrieved from the search using google scholar and PubMed. After removing the duplicate ones, the number of articles remaining was 643. Those articles were screened for title and abstract, and 529 were removed for not being relevant to this study or out of scope. The remaining 114 full-text articles were checked thoroughly, and 49 more were removed due to different reasons such as missing evaluation or comparison, insufficient dataset or sample number, lack of rationale behind decisions, insufficient explanation of experiment or method, etc. Finally, 65 articles were found relevant to this literature review and fulfilled the criteria in the eligibility section of the framework.

## 3. Traditional Techniques for BP Measurement

Among all the techniques, invasive and noninvasive arterial catheterization is the gold standard for accuracy. A thin and hollow tube is placed or inserted into the patient’s artery to perform this measurement technique. In the blood vessel, a manometer has been placed and used to measure the waveform of blood pressure change [52]. Blood pressure change can be tracked from the waveform and other cardiac activity [53]. This technique has been prevalent to frequently measure an ill patient’s BP reading (mainly in the incentive care unit). In case of hypotension, the patient can be given intravenous fluids to increase blood pressure immediately.

**Figure 2:**
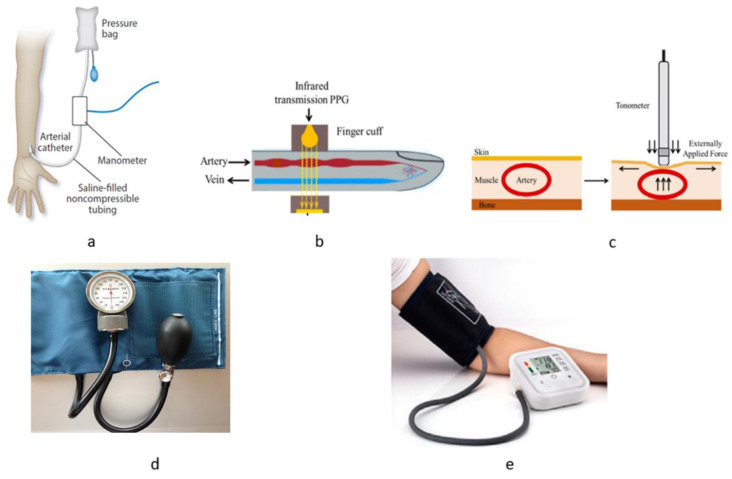
Traditional methods of BP measurement techniques. (**a**) arterial catheterization method, (**b**) vascular unloading technique, (**c**) arterial tonometry method, (**d**) auscultation method, (**e**) oscillometric method [19,43] CC by 4.0.

On the other hand, actions can be taken to lower blood pressure in the case of hypertension immediately. In both cases, frequent measurements are necessary to take urgent measures to avoid a more deteriorating conscience. The downside of these techniques is pain during the placement of the needle stick and catheter during insertion, potential infection in the place of catheters inserted, blood clots or bleeding during that time, etc. In Figure 2, traditional methods of noninvasive BP measurement techniques are shown. Among the noninvasive techniques for BP measurement, manual auscultation is an indirect technique where BP estimation is the most accurate [54]. This technique was initially invented in 1896 but later improved by Nikolai Korotkoff by placing a stethoscope over the brachial artery [55,56]. The blood flow sound disappears and reappears as the cuff is inflated and deflated. Using a sphygmomanometer, the measurement of SBP and DBP is done. The process requires an experienced clinician for reliable and consistent measurement [38]. Additionally, due to the toxicity of mercury in sphygmomanometers in many counties, the use of this tool is restricted. Additionally, due to the inflation of cuffs, considerable pressure is being put on the patient, which is not convenient for frequent measurement [57]. Among other BP measurement techniques, the oscillometric method is most common among manufacturers due to the emergence of digital electronics [58]. It measures mean arterial pressure and prepares estimates of SBP and DBP. The process starts with a cuff placed over the brachial artery in the upper arm; when the pressure increases through the cuff, the blood flow stops, and when the pressure releases, the pressure goes below MAP the pulsation decreases. From the oscillometric waveform in the time domain, the SBP and DBP were measured from MAP [38]. The downside of the oscillometric method is the uncertainty from the estimation of SBP and DBP, which does not reflect the standard expected from the Association for the Advancement of Medical Instrumentation (AAMI). There have been many variants of this technique using different signal analysis techniques, but still, the recommendation of AAMI is not met with consistency [59,60,61,62,63].

Using the modified Peaz principle, the vascular unloading technique is based on [64]. To maintain a constant blood volume, the pressure inside the artery is linearized by the outside pressure from the cuff placed on the finger [65]. The cuff consists of an infrared light source and photodiode to measure the blood volume in the arteries; the cuff’s pressure varies to maintain a constant blood volume. This intraarterial pressure is equal to finger cuff pressure determined by a manometer. Although continuous BP waveform can be generated with no chance of infection using this method, wearing a cuff for a long time is not convenient. Additionally, cuff size changes with finger size, and there is the possibility that finger pressure can be different compared to the considerable artery pressure making the measurement less reliable. Using arterial tonometry, a fair amount of pressure is applied on the finger over the radial artery to deform the shape of the finger [66]. The area where the measurement takes place should have a bone underneath the artery [67,68]; the pressure in the artery is proportional to the vertical displacement or bend in the finger [69]. The displacement is proportional to the arterial blood pressure waveform [70,71]. The shortcomings of this technique are a requirement of a specific type of place for measurement, and the position of that place must be very steady, making it unsuitable for more prolonged or frequent measurements. Additionally, placement accuracy is critical, etc.

## 4. Motivation for Cuffless Measurements of BP

This section discusses the motivation behind the cuffless blood pressure measurement. The discussion starts with the existing problem with traditional measurement techniques. After that, the current development of BP measurement devices will be summarized, and the rationale for research with the cuffless method must be examined. Additionally, the biomedical signals which are easily accessible and can be used for BP measurement in a cuffless manner will be discussed.

### 4.1. Limitations with Traditional Noninvasive Methods for BP Measurement

Although the measurement from the invasive method is direct from the blood flow in the artery, all the noninvasive measurement techniques are indirect. Thus accuracy and consistency are difficult to match. As per research, manual auscultation estimates the SBP lower than the invasive method and the DBP higher than the invasive method [72,73]. Many other techniques, such as oscillometric, auscultatory, and volume clamp devices, are calibrated using the invasive method. However, even with the calibration due to the chosen artery for measurement, the result still can be inaccurate [74,75,76]. Among all the conventional noninvasive methods, oscillometric devices dominate the clinical practice and the central portion of the database with BP measurement [77]. There are several limitations with the noninvasive methods based on the cuff. First, when it comes to the manual auscultation method, the cuff-based method requires trained professionals to perform the BP measurement accurately and reliably. Second, the cuff-based device is not readily available to everyone needing frequent measurements. Especially for the area where resources are limited, having an individual device for measurement is scarce, which is the main reason behind the awareness about own BP reading [78]. Third, during the measurement, the inflation and deflation of the cuff make inconvenience for the patient. Additionally, the patient must be still and relaxed during measurement, which is also tricky for frequent measurements [79,80]. Especially during nighttime, it is very cumbersome to have the cuff works frequently and hinders ambulatory measurement [81,82,83,84]. To solve these problems, the cuff must be removed from the setup while maintaining accuracy and reliability. The added functionalities needed are the ability to have frequent measurements with immediate results, a less cumbersome setup for measurement, the ability to provide user-friendly measurement techniques, and the inexpensive.

### 4.2. Necessity for Validation Protocol for Existing and Future BP Measurement Device

Among all the necessary clinical tests, measuring BP is one of the important ones, so both overestimation and underestimation severely impact the treatment process for the patient [85]. Usually, the most common error happens with overestimating BP measurement, which can cause issues such as wrong or untimely medications, unnecessary use of resources, and visits to physicians, etc. [86,87,88]. For example, hypertension prevalence impacts around thirty percent with a consistent inaccuracy of 2.7/5 mmHg [89]. Along with World Health Organization, several international organizations showed concern about validating the existing BP measurement device [90,91,92,93]. The main proposal was to strengthen the requirements for validation protocol and make it difficult for unvalidated products to be used or authorized to be sold. This proposal includes the device and new technology to measure BP, such as noninvasive cuffless methods. The World Health Organization (WHO) has presented the required specifications for automated noninvasive BP measurement devices with cuffs only; it encapsulates the ambulatory nature of measurement at home or outside and even in clinical setup [94]. The suggestions provided for BP measurement devices are, first, there has to be a solid regulatory board or office to ensure that only validated devices are available in the market [95]. Second, there must be a process for validating the device without bias. Third, manufacturers should disclose in detail the validation process they have performed before making the product available [95]. Fourth, the government should take necessary actions to make the validated product available to low resources areas and populations [54,96]. Fifth, to provide necessary training to professionals to differentiate between validated and not validated devices. Although the concerns from the WHO’s point of view are towards the cuff-based method, they have also targeted the research using the cuff-less method. Since some available devices work based on the cuffless method, those products should also be considered under the same validation protocol. Since all noninvasive methods are indirect estimations, validation should be critical while preparing or proposing any technique.

### 4.3. Improvement in Technology Helping Research with Cuffless BP Measurement

**Figure 3:**
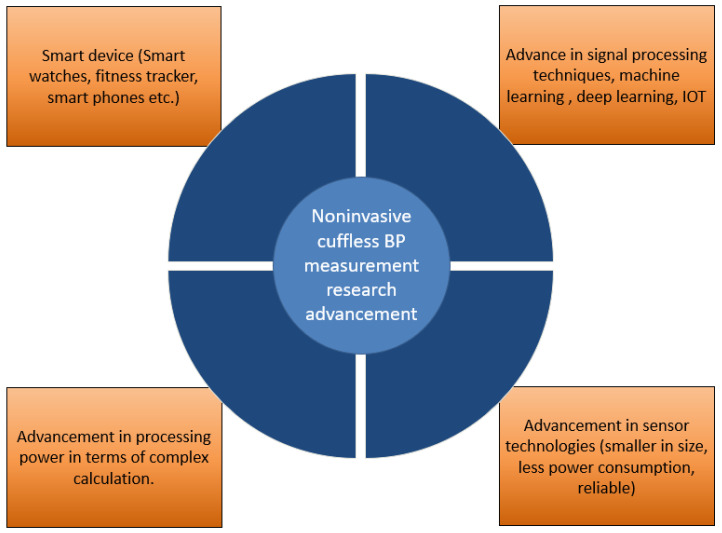
Technological advances behind the research progress in cuffless BP measurement techniques.

In this subsection, the emergence of the latest technologies enables the research of using biomedical signals to bring forth possible techniques to measure BP. Figure 3 depicts the technological advances responsible for the research progress in cuffless BP measurement techniques. The significant advancements are smaller sensors to acquire biomedical signals, improved signal processing techniques, highly efficient and robust calculation power, etc. [97]. First, the biomedical signals that are now being used in research will be discussed, then the adaptation of these sensors in convenient devices will be discussed. The ECG measures the heart’s electrical activity, which can be acquired by using electrodes placed in specific areas of the human body near the heart. A typical ECG wave consists of P, Q, R, S, and T peaks. The rhythmic activity acquired by ECG provides vast information about the structure and functions of the heart [98,99]. Different parts of the ECG wave can be correlated with the change of BP change after doing signal analysis techniques over the ECG wave [99,100]. Studies have shown the relation between changes in SBP and DBP with interval changes such as PR and ST segment [101,102].

**Figure 4:**
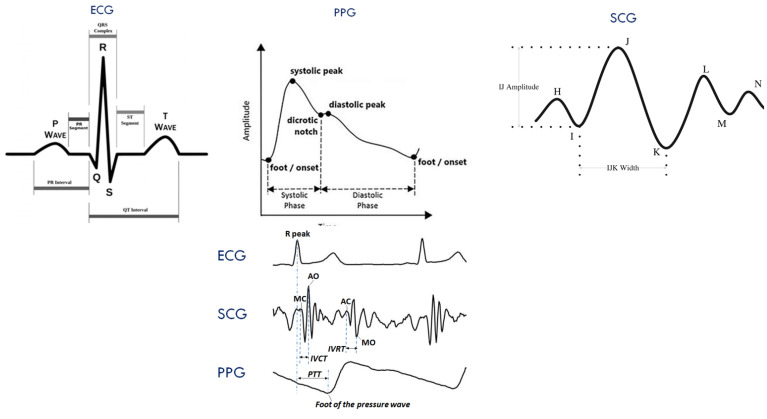
Biomedical signals used for recent BP measurement techniques (noninvasive and cuffless) [119] CC 4.0.

PPG signal provides the amount of oxygen saturation in blood using the calculation from transmitted and receiving light emitted from led in a specific area on the body such as the finger or ear [103,104]. Since blood pressure in arteries or blood vessels impacts the oxygen saturation or peak information or interval information from consecutive PPG waves, this signal has been extensively used in various research to find BP measurements [37,105]. When the blood is ejected from the heart, the body goes through changes such as displacement, acceleration, and these measurements can be recorded as ballistocardiogram (BCG) [106,107,108]. Figure 4 shows standard biomedical signals related to BP measurement techniques. Among other biomedical signals using ultrasound measuring absolute blood volume, blood volume oscillations measured by electrical bioimpedance and seismocardiography (SCG) measuring the lower frequency vibrations from the heart are also used in many research studies to make a relation with the change in blood pressure [108,109,110]. Due to the advancement in smartphones concerning a higher capacity for calculation and image processing ability—the smartphone can be used to create a PPG wave, and various information can be generated from that wave as a result [111,112]. Additionally, recent high-end smartphones have additional sensors to acquire different biomedical signals, such as SCG, PPG, ECG, and BCG, etc., to provide health alerts based on threshold strategies [113,114]. Like smartphones, smartwatches and fitness trackers are equipped with advanced sensor technologies and analysis capabilities to provide alerts for different health conditions [115,116,117,118]. To summarize, the recent technological advancement helped researchers have easy access to biomedical signals and high calculation capabilities in terms of speed and complexity.

### 4.4. Existing Wearable Device for Hypertension Management

The necessity of measuring blood pressure more frequently comes from excessive BP levels in the morning has been proven to be related to the risk of stroke, brain hemorrhage, and other organ damage [120,121,122,123,124,125,126]. Additionally, the nighttime elevation of blood pressure level is consistent with different cardiovascular issues [127,128,129,130]. So, the traditional habit of measuring blood pressure once in a while must be changed. Especially for people with a heart condition and stressful daily life, frequent measurement is a must to detect any deteriorating scenario beforehand. Compared to traditional BP measurement devices are cumbersome, the latest wearable devices can provide better adaptability for the patient. However, nearly all of the devices currently available in the market which are wearable and capable of measuring BP (as claimed by the manufacturer) are unvalidated and unreliable in terms of accuracy [95,131]. These facts did not stop the manufacturer from bringing devices with the ability to calculate BP from biomedical signals to some extent. In this subsection, only the devices currently existing will be discussed. Two devices from Omron healthcare have been submitted for validation criteria to AAMI; they fulfill the validation criteria while the patient stays sitting [132,133,134,135]. Another device from Omron was submitted for validation which works with higher accuracy while the patient remains in a specific orientation [133]. Both submissions were based on the device following an oscillometric measurement technique on the wrist. Other than those, some more devices tried to validate but did not match the threshold set by AAMI [135,136]. Two devices were submitted for validation to IEEE standards but did not pass the AAMI standards [137,138]. Additionally, till now, although there has been a significant number of studies done using PPG to measure BP, none of those made any substantial contribution towards consistent and reliable measurements of arterial BP [139]. Some devices combined PPG and ECG to make the measurement more robust, but even those did not pass validation with standardization organizations [140,141]. Some other approaches used other biomedical signals, such as bioimpedance with ECG but failed to uphold the criteria of standardization organization as a generalized rule [142,143]. Although wearable BP technology can potentially improve symptom detection for cardiovascular diseases, more research must be done before those can be used in real scenarios.

### 4.5. Rationale behind the Necessity to Continue Research on Cuffless BP Measurement

Research on cuffless blood pressure and measurement has recently increased rapidly [144]. At the same time, different devices claimed to be able to measure blood pressure using different types of biomedical signals are becoming available [144,145,146]. So, with the emergence of a high number of research and devices in the market, validating the techniques and devices is becoming increasingly important [74,147,148]. There are several standardization protocols for BP measurement, such as AAMI British Hypertension Society Protocol (BHS), and none were intended for cuffless blood pressure measurement [95,138,140,149]. So, until there is a universally acceptable validation protocol specifically for cuffless BP measurement methods and devices, it is not easy to accept the result or evaluation of techniques from any research, irrespective of how accurate or reliable the method claims to be.

Another problem is using a different number of samples and different kinds of datasets for training or testing the models. The validation protocol needs to provide specific information about evaluating the method, which can be compared or replicated easily [150,151]. Another challenge for the cuffless BP measurement techniques, which are calibration-free, is the significant variation that occurs in BP during the whole day due to lifestyle or daily work habits. Finally, most recent studies work with a trained model that compares different features with the change in BP. However, the actual relationship between the features and BP change is still unclear, especially for the techniques or devices that are not calibrated [152,153,154,155,156,157,158,159].

## 5. Features from Biomedical Signals for BP Measurement

During the last decades, numerous research has been done to find a new method for cuffless BP measurement, the rationale behind the choice of topic was the irritation and inconvenience caused by cuff based method. Additionally, the existing cuff-based method is unsuitable to have frequent measurements of BP. Among all the biomedical signals the scientist chose to work with, the most research involved photoplethysmography (PPG) signal [160]. In this section, the popular features from the biomedical signals that were used for cuffless BP measurement techniques will be discussed. The objective of the following discussion is not to summarize the research or articles producing a result but rather to summarize whether the results are consistent enough to be accepted.

### 5.1. Pulse Transit Time and Pulse Arrival Time

**Figure 5:**
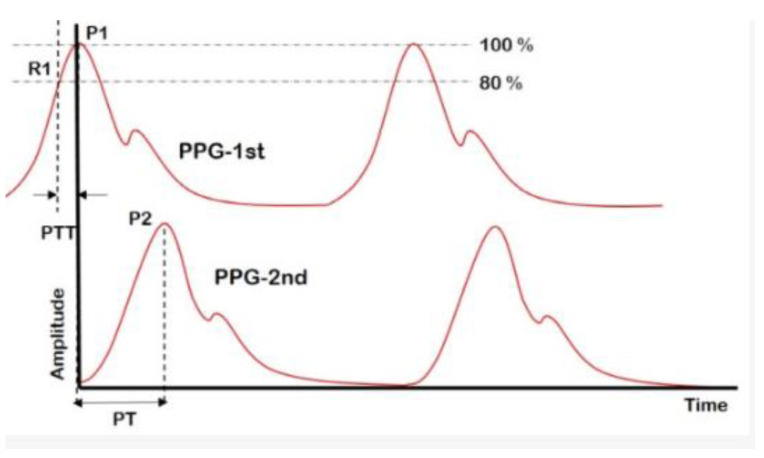
Example of pulse transit time [167] CC by 4.0.

Pulse transit time is defined by the time it takes for a pulse pressure wave to travel from one point of the artery to another, as shown in Figure 5 [36]. To measure the PTT, the most common method is to place two PPG sensors in two places close to the artery: ear, finger, toes, etc. [161,162,163]. Research has shown that there has been a reciprocal relationship between blood pressure and pulse transit time [164,165]. On the other hand, a researcher has also proved that only PTT is not sufficient to measure accurate BP levels [164,166].

Along with PPG, the ECG wave has also been used to measure PTT. In this case, measuring PTT means ECG is considered the proximal waveform, and another PPG sensor is used for the distal waveform to calculate PTT. Values of PTT from a specific patient and his/her BP can be related using a model based on empirical data. However, the problem is that the data needs to be calibrated frequently due to vascular aging. The correlation between PTT and BP levels is good but not strong enough to have a conclusive result; mostly, the correlation value remains between 0.7–0.8 [168,169,170,171]. Till now, there has been no proof of any PTT-based method which can be used as a general formula or method for BP measurement, the most common flaw is that once the model is evaluated using a dataset other than the one used for producing the technique, the evaluation fails to uphold any standard protocol (AAMI or BHS) [172,173].

Similar to PTT, there is another parameter that is popular among researchers is pulse arrival time. It is the time difference between heart activation and pulse wave at a distal point such as a finger. PAT can also be defined as pre-ejection delay plus the PTT [36]. Usually, the peak of the ECG wave is used as the proximal point, and another PPG sensor is a distal point to measure PAT [174,175,176]. Similar to PTT, the PAT is also influenced by different physical attributes of the patient and shows similar limitations using it in the model for BP measurement [172,173]. Studies proved PAT to be better-suitable than PTT but require calibration for individual use.

### 5.2. Pulse Wave Velocity

Pulse wave velocity (PWV) is the pulse wave’s velocity that travels from a proximal point to a distal point through a length L [29,177]. The relation between PTT and PWV is reciprocal. Because the elasticity of the artery changes from person to person, even with the age of the same person, the length of L will also change from person to person. Despite these issues, researchers tried to find the relationship between PWV and BP levels (SBP and DBP) [178,179].

$$\mathrm{PWV}=\frac{L}{\mathrm{PTT}}=\sqrt{\frac{\mathrm{artery}\;\mathrm{wall}\;\mathrm{thickness}\times \mathrm{Elasticity}\;\mathrm{of}\;\mathrm{artery}\;\mathrm{wall}}{\mathrm{blood}\;\mathrm{density}\times \mathrm{distance}}}$$

The relationship can be illustrated using the Moens-Kortweg equation, as shown above [180]. The existing proposed techniques require extensive calibration, even for a single person. Since the model scientist produced can not be generalized, and the calibration requirement frequently uses a cuff-based method, no clinically usable device or end product is available based on PWV [181,182].

### 5.3. Pulse Wave Analysis

**Figure 6:**
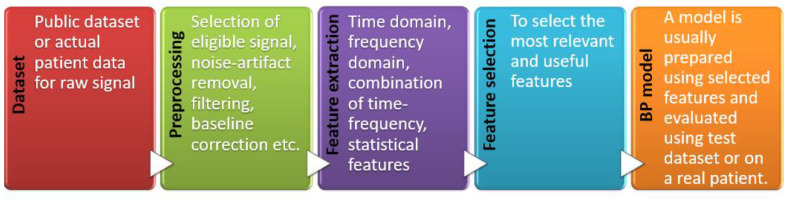
Typical steps for pulse wave analysis to get BP measurement model.

**Figure 7:**
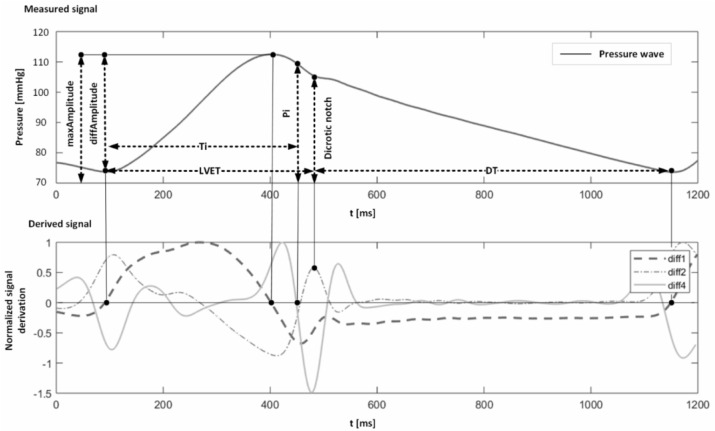
Example of features in pulse wave analysis [186] CC by 4.0.

Pulse wave analysis means using the pulse wave through signal processing techniques and extracting necessary features for further use in building a model for BP measurement. A typical step for pulse wave analysis to get a BP measurement model is shown in Figure 6. The process requires one or more sensors to measure PPG waves, signal processing tools for removing noise and artifacts, feature extraction and selection, etc. Many attempts have been made to build a model for BP measurement using pulse wave analysis [159,183,184,185]. Examples of features in a typical PPG wave are shown in Figure 7.

**Figure 8:**
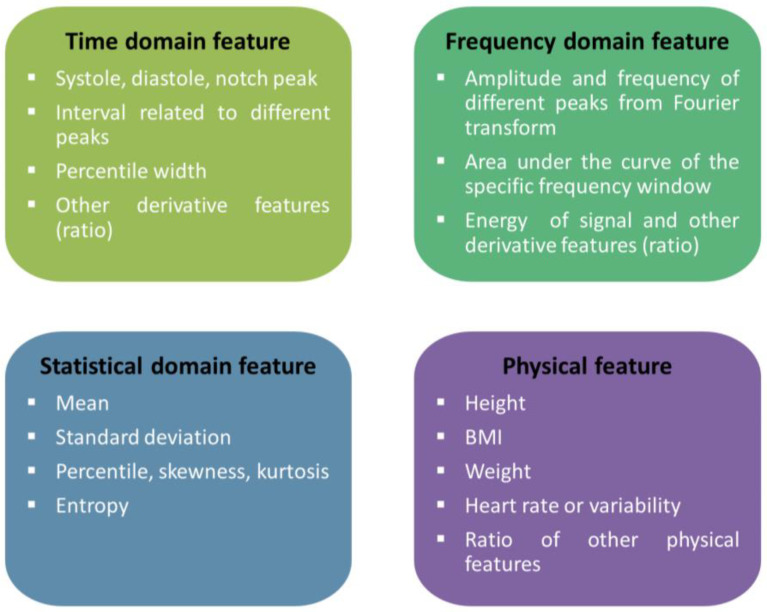
A summary of features used from biomedical signals (typically PPG wave) to create a model for BP measurement (cuffless-noninvasive).

Four main types of features are popular among researchers to extract from pulse waves for cuffless BP measurement: time domain-based, frequency domain-based, time-frequency domain based, and statistically based, as shown in Figure 8. Some researchers opted for single-domain features, while others opted for a combination of several domain features. A typical pulse wave analysis diagram is shown in Figure 6. Processing raw signal for noise or artifact are not in the scope of this literature review; rather, the focus will be the typical features used by the researchers from pulse wave analysis to create a model for BP measurement. As shown in Figure 7, a common strategy is to get the derivatives of the PPG signal to get different peak values and intervals. Additionally, using the Fourier transform, the primary information can be achieved at significant frequencies. Other than the physical features such as age, height, weight, BMI, heart rate, etc., another aspect researchers follow for features are statistical features [187,188,189]. The time domain, frequency domain, statistical domain, and physical features used in the literature survey have been summarized in Figure 8 [190,191,192,193,194,195].

## 6. Machine Learning and Deep Learning in Cuffless Noninvasive Blood Pressure Measurement

This section will discuss machine learning and deep learning in the studies and research to measure cuffless noninvasive blood pressure. The focus of the discussion will not be on how individual techniques proposed by the researcher performed but on how and which algorithms were implemented and their respective shortcomings. The rationale behind the choice of focus is, comparing or summarizing performance will not add any value since there is no general protocol available for validation of the technique for cuffless noninvasive BP measurement. So, the literature survey will be more effective and informative if it involves how the algorithms were used.

### 6.1. Conventional Machine Learning Algorithms

Conventional machine learning algorithms involve manual extraction and feature selection. Starting with the raw signal, preprocessing the signal, extraction of features, and selection of features, the finalized features are used to train the machine learning algorithms to create a model. The most popular machine learning algorithm used widely is the regression algorithm [189,196,197,198,199,200,201,202,203]. The regression algorithm can be in several forms: linear regression, least square regression, lasso or ridge regression, etc. To summarize how the regression algorithms were implemented, most studies used a PPG signal or a combination of PPG and ECG signals to extract the required features from those signals. After getting features, the usual process was to take the most valuable and relevant features and use only those to train the regression model. Later the regression models were evaluated using the same dataset. Nearly all the studies followed this same basic format; the changes from one study to another were mostly in the choice of signal, several features to be extracted, the algorithm used to optimize features, and the sample number for training and testing. The typical limitations found were the inability to reproduce performance with a separate database other than the one used for training a small number of samples to train with. The way forward for regression-based analysis is to use a large dataset, and more research needs to be done before claiming to achieve any significant and usable outcome. Similar to regression algorithms, the research used a support vector machine (SVM), AdaBoost, random forest, and K-nearest neighbor, etc., to find a model to measure BP [185,204,205,206,207,208,209,210,211]. Additionally, the limitations were almost the same for these conventional machine learning algorithms. Although some studies claimed that one of the conventional machine learning algorithms performed better than others, their performance was not up to the mark or consistent regarding following standards such as AAMI or BHS. Based on the literature survey, it is impossible to agree with any claim about the superiority of any conventional machine learning algorithm over others.

Although numerous publications have used conventional or shallow machine learning architecture to find models to measure BP, none of these came into reality or were of clinical use. If researchers want to pursue this path for a solution, they need to make sure to do the following to make their solution sustainable and reliable:

- The model must reproduce the claimed result using other datasets or real-time data.
- The rationale behind the performance should accompany the use of algorithms.
- The number of features and optimization algorithms needs to be explainable.
- AAMI and BHS standards need to be passed for both SBP and DBP.
- Since all the features are from biomedical signals due to physiological activities, calibration is mandatory. The purpose, frequency, and procedure of calibration need to be addressed.
- The database (either online or real patient data) needs to be well distributed regarding demographic data.
- There has to be a generalized validation protocol in place so that the performance of all the techniques can be compared without any bias.

### 6.2. Deep Learning Algorithms

**Figure 9:**
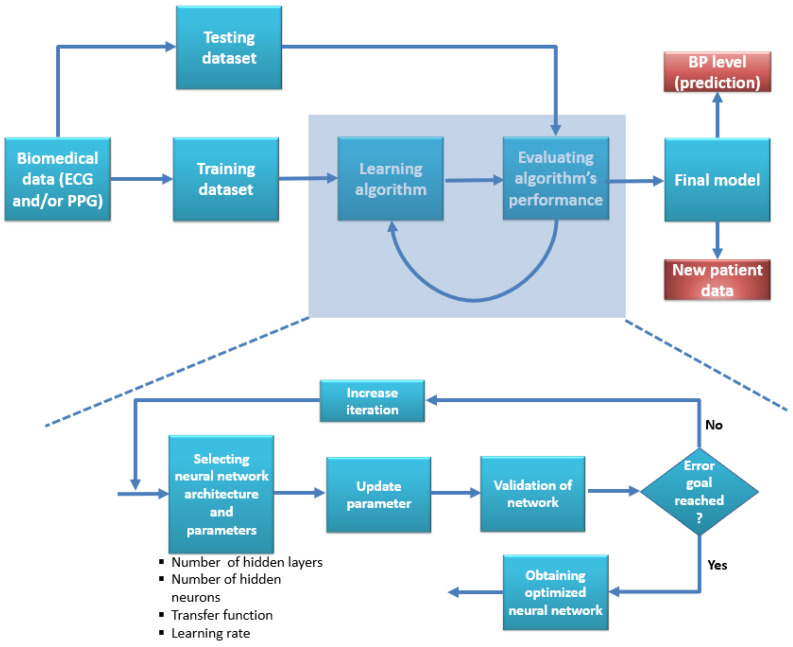
An example of a deep learning network flow diagram.

With the advancement of deep learning networks through artificial neural networks (ANN), convolutional neural networks (CNN), recurrent neural networks (RNN), Long and short-term memory (LSTM), etc., they are using different biomedical signals such as ECG and PPG into learning network to achieve a model for BP measurement becoming very popular among scientists. The main reasons are: compared to shallow machine learning algorithms, deep learning algorithms can extract features on their own and can do a better job in most cases by finding features that might have been missed in the case of shallow ML, also although it is computationally extensive the performance achieved from deep learning networks were much better than conventional ML algorithms. For the past few years, scientists have tried every possible deep-learning algorithm for BP waveform prediction, where the model automatically learns critical features. The ECG signal can be acquired using an electrode over a specific part of the upper torso; the PPG signal can be acquired from either ear or fingertip. The gold standard arterial blood pressure can be chosen from the cuff-based method or a radial artery catheter. Figure 9 depicts an example of a deep learning algorithm from start to finish.

Although preprocessing is a common step for any machine learning algorithm, there is always room for improvement during preprocessing steps, which can make an evident difference in the outcome of the model’s performance. An example of a flow diagram of a deep learning network is shown in Figure 9. Since the deep learning algorithm takes care of the feature extraction and assigning appropriate weight for the impact of each feature, the preprocessing step needs to ensure the raw data is free from noise, segmented for training; inputs are normalized, etc. Due to the temporal similarities between PPG and ABP, most studies have used PPG as an input signal for their respective deep-learning algorithms. With the invention of new deep learning algorithms, researchers have tried almost all the available methods, such as wavelet neural network, long short-term memory, ANN, U-Net, MultiResUnet, V-Net, GAN, etc., for BP measurement model [24,32,35,212,213], even some studies involved using a hybrid model where multiple algorithms were used together for better performance [10,35,214,215,216,217,218,219,220,221,222,223,224]. A study was conducted to measure blood pressure by combining information from waveform (ECG and PPG), patient physical information, and features such as PTT and passing then as input of a neural network [225]. They have found that using features combined with deep learning algorithms worked better than using neural networks alone. Since all these studies were data-driven and the deep learning model follows a black box-themed approach, the only way the advantage or disadvantages can be discussed is based on input data processing and output data performance. The most common disadvantage of using deep learning networks was extensive computational complexity, lack of rationale behind how and why the architecture worked, a small number of subjects, use of the same dataset for training and testing, etc. On the plus side, nearly all those algorithms performed better than the conventional shallow machine learning algorithms. Compared to the scenario where shallow machine learning algorithms failed to pass the AAMI and BHS threshold, there are cases in recent years where the use of deep learning algorithms resulted in a ‘pass’ in AAMI or grade “A” or “B” in BHS standards [24,33,34,213,226].

In this section, the summarization or discussion of the individual study was intentionally avoided due to the following facts:

- Although deep learning models provided better accuracy compared to shallow machine learning algorithms, the number of studies that matched the AAMI and BHS standards is deficient.
- Since the approaches are mainly data-driven, the performance using the author’s choice of the dataset for their model should match using another random dataset. Nearly all the studies used the same dataset for test and validation.
- Since deep learning algorithms require large samples to be adequately trained, there is a high chance that all the experiments using microscopic subjects may result in overfitting the technique.
- There is no general validation approach available to compare performance among different studies.
- No clinically acceptable method was produced or implemented to have a gold standard using deep learning techniques.

Although deep learning network does not require the manual intervention of feature extraction or feature optimization, these models are heavily computationally extensive. On the other hand, due to the lack of a global standard dataset, experiment protocol, or validation protocol, this research area is now limited to plugging biomedical signals, especially PPG and ECG, into different types of deep learning algorithms and their hybrid versions.

## 7. Dataset Used for BP Measurement Model

Among all the databases used in studies to make machine learning models for cuffless noninvasive BP measurement using biomedical signals, the most common ones are the University of Queensland’s vital signs dataset [227] and MIMIC [228]. Since deep learning requires a much larger dataset, most of the dataset is used for shallow machine learning algorithms, and nearly all private datasets can not be used for deep learning algorithms due to being too small. The following table contains the most popular database, the number of data, the signal from the database, and a short description. Since there is no general guideline or protocol available to create a database for the BP measurement model, the database has some disadvantages coming along with it. Table 1 shows different datasets being used in BP measurement models.

## 8. Challenges and Future Recommendations

The continuing research to get cuffless and noninvasive BP measurement model are facing challenges such as data collection, the accuracy of control data, standardization of public dataset, need for validation protocol, patient-specific issues, calibration, the efficiency of the algorithm, integration with the traditional method, issues with the specific dataset, deployment issues, and collaborations, etc. Along with discussing the challenges, there will be recommendations and ways forwards for the researcher to pursue their investigation.

### 8.1. Data Collection and Accuracy of Control Data

Until now, there is no standardized protocol for data collection to use the studies to make a BP measurement model. Different experiments have used different protocols while collecting data; on top of that, the dataset extensively used in research also employed different protocols. Additionally, if the objective is to prepare a model to be used by general populations of all ages, the training data needs to be collected, keeping that in mind. Most of the online dataset was collected during the patient’s intensive care unit stay, which can be impacted by many variations such as drug, type of disease, etc. Additionally, data acquisition should be made in a stationary position since any movement may add noise and unnecessarily change the rhythm. The protocol should be implemented so that, initially, the learning should start using static data, and gradually the learning should adapt to more dynamic situations.

### 8.2. Validation Protocol

There have been a significant number of studies done on this topic. However, due to a lack of similarity between the dataset, chosen data sample, or data acquisition protocol, it is tough to compare one technique. Some of the techniques are mathematical equation based, and some are machine learning algorithm based; the result from both may claim higher accuracy, but there is no way to compare which algorithm performed better. While working with machine learning algorithms, popular datasets contain ABP waveform to consider as the gold standard. When the dataset is privately owned or created from real-life patients, the number of subjects usually remains very small, and at the same time, getting ABP is difficult. To solve this problem, there must be a documented protocol for acquiring data from the patient, selecting the patient, preprocessing the data, and testing the data after preparing the model. Till now, there has been no such protocol available. Additionally, even in the successful experiments where a specific algorithm performed well, none of these have gone through a clinical trial. So the research speed needs to be matched with making the innovation available to be used clinically.

### 8.3. Calibration

One of the most difficult challenges for the current research work in this area is to produce a standard calibration approach that will not only provide a person-specific reliable BP model but also get updated to continue providing results with similar accuracy in the future. Researchers have tried to use a large dataset with sufficient variation and a wide range of data to train the model. However, no attempt replaced the necessity to calibrate using a standard method from time to time. There have been several attempts to calibrate the model, or in other words, fine-tun the parameters; such an attempt was with PTT calibration using data every 24 h using photoplethysmography intensity ratio [234], some other calibration attempts involved sphygmomanometer data [235,236]. However, none of the calibration methods is practical to implement or remove the need to use standard gold measurement systematically. The problems are twofold. First, a calibration need is due to a person’s physiological change. Without calibration, different health parameters’ impact on blood pressure change will go unnoticed. Second, a model prepared for a specific group of patients’ data can not be used as a general protocol unless it contains a calibration segment that adjusts the model parameter for another group of persons. So, preparing a model is only a part of the solution; when it comes to making the model usable clinically for the mass population, there has to be a standardized solution, including calibration steps that will not require the patient to use the cuff-based method frequently.

### 8.4. The Model or Technique Needs to Be Interpretable

Artificial intelligence in machine learning and deep learning dominates the current research endeavors regarding noninvasive cuffless BP measurement. With the advance of deep learning algorithms, more and more studies are getting published with more advanced deep learning models and better accuracy. However, there is a considerable gap to fill when explaining the necessary knowledge to elaborate on the model and its relation to the result. Without a proper explanation of the black box’ nature of the deep learning model to the audience, such as clinicians, physicians, health care workers, etc., there will always be an issue of trust. The researchers need to understand the necessity of creating a model that can be explained with a rationale to the people who will use the model confidently. Interpretability is a significant part of diagnosis and decision-making in the medical arena. Healthcare professionals must explain or at least understand how and why the model produces any specific result. Since feature selection approaches can be easily explained and related to medical professionals’ biological knowledge, researchers need to look for either keeping the deep learning model simple or using a hybrid model.

### 8.5. Model Deployment

The objective is to let the patient measure frequent blood pressure. However, the day is convenient, so using the small and portable device is mandatory to ensure the measurement setup is not cumbersome and hinders daily activities. Now, the majority of the research available only concerns themselves with only preparing a model and the performance of the model. However, very few tried or designed a small system that can replicate the model in real scenarios or work for actual people. The main challenges of deploying the model into real-world applications are. First, the signal acquisition in research mostly happens in the patient’s controlled environment and posture, which will not be the case in real life; second, the small and portable devices need the necessary calculation power to deliver accuracy and calibration capacity frequently. All the devices that can use the biomedical signal to measure BP are not certified or acceptable in clinical environments, so once researchers advance to deploying the models into the device, the challenges will come in a different form. Researchers may consider the challenges of possible deployment ahead of designing the model.

### 8.6. Necessity of Collaboration with a Health Professional

The research field needs collaboration between machine learning, signal processing expert, and health care professionals. To tackle the three main fronts of the solutions: acquiring proper signals, preparing a model, and deploying the model into the device, the collaboration can make a better plan. Right now, the majority of the studies are done involving machine learning experts only. The problem arises when the model’s interpretability and deployment come into question. The signals can be mixed with different kinds of noise, artifacts, and motion in an accurate word scenario compared to a lab scenario. Additionally, when it comes to deployment, the end device needs to be adaptable to the model and the calculation capacity needed by the model. Additionally, irrespective of the performance of the device or model, the professional medical needs to be confident about the result since they make the decision that can either improve or deteriorate patients’ health condition.

## 9. Conclusions

This work reviewed current development in cuffless and noninvasive blood pressure measurement techniques using biomedical signals. More specifically, the features from signals, machine learning, and deep learning were the main focus of this literature review. Although a significant amount of research happened with advanced signal processing and machine learning techniques, there are still scopes of improvement in consistency, calibration, interpretability, collaboration with medical professionals, deployment into a user-friendly device, etc. Since there is no device available now certified by an accepted regulatory body to be used clinically, the individual performance of the research was not the main focus of the survey. Instead, the focus was on the reasons behind the shortcomings and challenges. Unless the performance of the models developed by researchers using either an online dataset or a private dataset created manually can be replicated in an actual application and on a new patient, the goal of the research is to let people measure BP more frequently without cuff can not be materialized. Several recommendations and future research directions have been provided, such as: building an acceptable universal dataset for training models, making calibration without using cuff based method, creating a real-life scenario to account for all kinds of noise while acquiring signals, planning deployment feasibility in the research plan, making the models interpretable to physicians to gain confidence, etc. Unless these research paths are ventured extensively, the cuffless noninvasive BP measurement model using biomedical signal analysis should not be used in a clinical setup.

## Figures and Tables

**Table 1:** Datasets used in different BP measurement models.

Dataset	Data	Signal	Description	Reference Research
MIMIC II [229]	26,870	PPG	It contains different physiological signals, including blood pressure, PPG, and ECG. There is confusion about whether the data were synchronized, so using MIMIC II for any data such as PAT is not recommended.	[230]
MIMIC III [228]	40,000	ECG, PPG	It is a collection of physiological data from a different hospital; most research with clinical data for the BP measurement model has used this dataset.	[231]
University of Queensland’s vital signs dataset [227]	23,617	PPG	This dataset includes blood pressure measurement along with PPG signal. The case number is limited compared to MIMIC II and III.	[218]
SHAREE (Smart Health for Assessing the Risk of Events via ECG) [232]	24 h electrocardiographic (ECG) Holter recordings of 139 hypertensive patients	ECG	Anti-hypertensive treatment was given to 139 patients, and after one month, the ECG Holter was used to record data. Each recording was of 24 h, containing three ECG leads sampling at 128 Hz.	[233]

## Data Availability

Not applicable.
